# Correction to “Long‐Read Sequencing of CAH and ADPKD Provides Novel Insights Into the Genetic Diagnosis of Male Infertility”

**DOI:** 10.1002/rmb2.70071

**Published:** 2026-06-22

X. Li , C. Liu , W. Liu , et al., “Long‐Read Sequencing of CAH and ADPKD Provides Novel Insights Into the Genetic Diagnosis of Male Infertility,” Reproductive Medicine and Biology 25, no. 1 (2026): e70038, 10.1002/rmb2.70038.41937974 PMC13045334

The first affiliation should be corrected as follows:

Xiao Li^1^ | Chang Liu^2^ | Wen Liu^1^ | Xianlong Wang^1^ | Jiaolong Liu^1^ | Feifei Cai^1^ | Shaoming Lu^1,3^

^1^State Key Laboratory of Reproductive Medicine and Offspring Health, Center for Reproductive Medicine, Institute of Women, Children and Reproductive Health, Shandong University, Jinan, Shandong, China | ^2^Berry Genomics Corporation, Beijing, China | ^3^Center for Reproductive Medicine, Shandong Provincial Hospital Affiliated to Shandong First Medical University, Jinan, Shandong, China

We apologize for this error.

